# COVID‐19 and misinformation: Is censorship of social media a remedy to the spread of medical misinformation?

**DOI:** 10.15252/embr.202051420

**Published:** 2020-10-26

**Authors:** Emilia Niemiec

**Affiliations:** ^1^ Centre for Research Ethics and Bioethics Uppsala University Uppsala Sweden

**Keywords:** S&S: Economics & Business, S&S: Ethics

## Abstract

Social media companies have resorted to censorship to suppress misinformation about the COVID‐19 pandemic. This is not the most prudent solution though given the uncertainties about the disease.

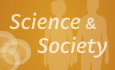

In February this year, when the new coronavirus began to spread outside China, the Director General of the World Health Organization (WHO), Tedros Adhanom Ghebreyesus, announced: “we're not just fighting an epidemic; we're fighting an infodemic” (https://www.who.int/dg/speeches/detail/munich-security-conference). The term, coined in 2003 in the context of the first SARS epidemic, refers to a rapid proliferation of information that is often false or uncertain (https://www.merriam-webster.com/words-at-play/words-were-watching-infodemic-meaning). Academic researchers, international organizations such as the United Nations and the European Union, individual governments and the media have acknowledged and discussed the prevalence of the alleged COVID‐19 infodemic and the importance of fighting it. Information campaigns have been launched to provide wider audiences with reliable information about COVID‐19. Main social media platforms have also actively fought against false information by filtering out or flagging content considered as misinformation. In this essay, I will discuss the censorship on social media platforms related to COVID‐19 and the problems it raises along with an alternative approach to counteract the spread of medical and scientific misinformation.

## Censorship on social media platforms

Censorship on major social media platforms, such as Facebook, Twitter and YouTube, is not a new phenomenon. These companies regularly remove content that they consider as objectionable based on continually updated categories outlined in their policies. Examples of “objectionable content” include “hate speech”, “glorification of violence” or “harmful and dangerous content”. These categories are not only often broader than the exceptions to the freedom of speech entrenched in legislations of democratic countries, but also implicitly vague and leave plenty of room to interpretation. Indeed, an analysis of content banned on social networks suggests that the moderation is often politically biased (Stjernfelt & Lauritzen, 2020). Some very recent examples of moderation with apparent political ramifications include Twitter's labelling of US President Donald J. Trump's tweets as violating Twitter's policy about glorifying violence or abusive behaviour, or adding a warning suggesting that a post was factually inaccurate (https://twitter.com/realdonaldtrump/status/1275409656488382465; https://twitter.com/realDonaldTrump/status/1266231100780744704; https://twitter.com/realDonaldTrump/status/1265255835124539392).

… an analysis of content banned on social networks suggests that the moderation is often politically biased …

Social media platforms are private companies and as such, one could argue, they should be able to decide what content they tolerate or not. However, such a view overlooks salient aspects of the issue. First, censorship on Facebook, Twitter or YouTube appears to contradict the very idea of these communication networks, that is, of spaces where everyone can express their opinion. YouTube, for example, declares on its website that its “mission is to give everyone a voice” (https://www.youtube.com/about/). Twitter's manager once described his company as “the free speech wing of the free speech party” (https://www.theguardian.com/media/2012/mar/22/twitter-tony-wang-free-speech). Mark Zuckerberg, the CEO of Facebook, has similarly been vocal about Facebook's commitment to the freedom of speech (https://about.fb.com/news/2019/10/mark-zuckerberg-stands-for-voice-and-free-expression). Many users of social media might have believed in these ideals when joining the online communities. Or, at least, they did not expect biased censorship on the platforms. From this point of view, appeals to the freedom of speech made by the social networks seem unfair or deceptive.

Another important point to consider is the fact that a few big tech companies currently dominate social media services, which also serve as a source of news to many users. According to a 2020 survey by the Reuters Institute for the Study of Journalism, 36% of 24,000 respondents from 12 countries use Facebook for news weekly, while 21% of the surveyed use YouTube for the same purpose (https://reutersinstitute.politics.ox.ac.uk/sites/default/files/2020-06/DNR_2020_FINAL.pdf). If we add to this the fact that Google is the most popular search engine, it becomes clear that a few tech companies have huge power over what information Internet users can see and how their views are shaped.

Referring among others to the role of the “modern public square” ascribed to online platforms, President Trump recently issued an executive order which aims to limit the current legal protections of the big tech companies and prevent the censorship on their platforms (https://www.whitehouse.gov/presidential-actions/executive-order-preventing-online-censorship/). What will be the effect of the order and whether it is an adequate solution to the problem is yet to be seen. What is clear, however, is that the tech giants’ role in shaping public discourse has become apparent. The governments of various countries have either attempted to find a solution to this issue or to use the possibility of censorship by big tech companies for their own purposes (Stjernfelt & Lauritzen, 2020).

While it is difficult to overlook the politically motivated censorship on online platforms and its implications, the removal of misinformation related to medical topics such as COVID‐19 may seem to belong to a different category—not political, but rather one of science, where information can be objectively judged based on scientific evidence. At a closer look, however, this does not seem to be the case.

… censorship on Facebook, Twitter or YouTube appears to contradict the very idea of these communication networks, that is, of spaces where everyone can express their opinion.

## Censorship of information about COVID‐19

In response to calls to combat misinformation about COVID‐19, a group of companies, including among others Facebook, Twitter and YouTube, issued a joint statement in mid‐March this year. They stated that they are “jointly combating fraud and misinformation about the virus, elevating authoritative content on [their] platforms” (https://about.fb.com/news/2020/10/coronavirus/#joint-statement). Their actions include the introduction of “educational pop‐ups connecting people to information from the WHO” (Facebook), adding warning labels to content considered as false or misleading (Facebook, Twitter), removing content contradicting health authorities or the WHO (YouTube) and content that could directly contribute or lead to (physical) harm (Facebook and Twitter) (https://about.fb.com/news/2020/03/combating-covid-19-misinformation/, https://covid19.twitter.com, https://support.google.com/youtube/answer/9891785). One example is a video removed by YouTube, in which a researcher, John Ioannidis, discussed data related to COVID‐19, questioned the need to continue the ongoing lockdown and raised concerns about the negative impact of the restrictions (https://medium.com/@michaelaalcorn/how-wrong-was-ioannidis-5940e49c9af6). Other cases of censorship on major social media platforms have been reported, for example removal of information about anti‐quarantine protests on Facebook (https://www.reuters.com/article/us-health-coronavirus-usa-facebook/facebook-removes-anti-quarantine-protest-events-in-some-us-states-idUSKBN2222QK).

A major question regarding the policies of the communication platforms is who exactly defines and how which information is deemed to be false or harmful? And can we rely on these judgements? One of the authoritative sources that all three major social media platforms mention in their policies on COVID‐19 is the WHO. It is an established and influential organization, yet it may make mistakes, including in the context of handling epidemics. For example, concerns have been raised about influences of pharmaceutical companies on the guidelines related to the flu pandemic in 2009 (Cohen & Carter, 2010).

A major question regarding the policies of the communication platforms is who exactly defines and how which information is deemed to be false or harmful?

YouTube and Twitter also refer to guidelines from local health authorities. Although these are usually developed by experts and may be legally binding, this does not imply that they are unerring. There has been disagreement among researchers in medical sciences about the necessity for lockdown measures (Melnick & Ioannidis, 2020). Furthermore, researchers and many healthcare professionals have indicated numerous and serious negative impacts of the policies introduced to combat the spread of COVID‐19, and expressed doubts about the evidence supporting these measures (Ioannidis, 2020) (https://www.scribd.com/document/462319362/A-Doctor-a-Day-Letter-Signed).

This variety of opinions on how to handle the COVID‐19 pandemic is related, among others, to the fact that it is a new disease and the knowledge about it is relatively limited and unsettled. Moreover, the implications of the pandemic and measures taken to counteract it exceed the remit of epidemiology or public health experts and fall into areas of economy, education, psychology and sociology. Meanwhile, experts who develop policies or express opinions about COVID‐19 may not have a complete overview of the implications of pandemic‐related policies.

Processes of reviewing research results, drawing conclusions, and preparing guidelines may be complex, prone to mistakes and not immune to political or commercial interests.

Additionally, there are the “usual” problems related to evaluation and translation of evidence into medical or public health practice. They include questions about the validity of a given study, limitations of methods, reproducibility of results and so on. Processes of reviewing research results, drawing conclusions and preparing guidelines may be complex, prone to mistakes and not immune to political or commercial interests. Retracted articles on COVID‐19, including publications in *The Lancet* and the *New England Journal of Medicine* (https://retractionwatch.com/retracted-coronavirus-covid-19-papers/), suggest that research on COVID‐19 is not an exception to problems related to the ethics of research.

Constructive critique, questioning of evidence and opinions of scientists and policy‐makers are thus necessary to identify and correct potential errors and to prevent them from being propagated. By following their policies on COVID‐19, social media platforms filter out content which contradicts specific views that are not necessarily correct or unanimously accepted, with respect to the underlying scientific evidence or represented values and political views. If critique of these views is eliminated or restricted, the possibility to correct errors, contribute to the understanding of the topic and inform public debate is limited. Additionally, since the censorship is not based solely on science—as scientific evidence is currently limited and medical experts still disagree on various topics—other factors influence decisions to remove content. Questions about the commitment to the freedom of speech of the social media providers and risk of manipulation of public opinion are therefore relevant also in case of information about COVID‐19.

## The remedy to medical misinformation

If censorship of scientific information does not seem to be an adequate solution to the problem of false medical news on social media, what then is a fitting remedy to the “infodemic”? In order to adequately address this question, it seems that a few related and more fundamental issues should be addressed. What exactly is the COVID‐19 infodemic? Based on what criteria is it declared and what are the implications of such a declaration? How do the different actors define “misinformation”? What are the actual and potential harms of the spread of false medical information? These questions should be answered in order to determine what exactly the problem is that we are trying to solve.

General understanding of how social media function may help users make informed decisions about the use of Facebook, YouTube, Twitter and similar services …

Notwithstanding, we may reflect on general approaches to prevent the potential harms related to misinformation. Education and raising awareness among publics, including during formal school education, may be one crucial strategy in immunizing society against misinformation. In this context, two areas of knowledge appear particularly relevant for Internet users: related to social media, in particular, the mechanisms they use, their business models, as well as benefits and risks related to the use of their services; and general knowledge related to science reporting, scientific research and its limitations.

General understanding of how social media function may help users make informed decisions about the use of Facebook, YouTube, Twitter and similar services, in accordance with one's goals and values. In particular, the fact that social media platforms are provided and operated by private companies, which are interested primarily in making profit, and the implications of this fact may be worth to consider. The business model of social media companies is based on revenue from ads tailored to the users: the more users and the more time they spend on their websites, the higher the profit. Consequently, these companies use knowledge from psychology and huge amounts of personal data to design ever more efficient mechanisms to motivate users to spend more time on their websites. Sean Parker, a former president of Facebook, put it this way: “…we need to sort of give you a little dopamine hit every once in a while, because someone liked or commented on a photo or a post or whatever. And that's going to get you to contribute more content, and that's going to get you… more likes and comments. It's a social‐validation feedback loop … exactly the kind of thing that a hacker like myself would come up with, because you're exploiting a vulnerability in human psychology.” (https://www.axios.com/sean-parker-facebook-was-designed-to-exploit-human-vulnerability-1513306782-6d18fa32-5438-4e60-af71-13d126b58e41.html).

The human psychology used by social media sites—for example the need of social approval, reciprocity and novelty seeking—may also play a role in the spread of misinformation. For example, a study of news shared on Twitter suggests that not only false stories are more likely to be shared than true news, but also that false news is usually more novel than the true one (Vosoughi *et al*, 2018). Realization of the psychological mechanisms that often drive the use of social media and sharing information as well as of the benefits gained by the companies from the use of their platforms may help avoid being manipulated and prompt more reflection over why we share or interact with a given content.

Furthermore, better awareness of the variable quality of science reporting as well as the limitations of scientific research may be helpful to discern whether one should share a given science news story or medical advice. General public usually receives science news from media, notably newspapers and blogs where reporting may be, intentionally or not, biased or erroneous. Moreover, even if science reporting is of highest quality or one reads directly scientific articles, one should keep in mind that there is still a risk of mistakes or bias. Research is being conducted by people who are not free from mistakes, career or commercial interests, political and moral views, and other influences which may impact their conclusions. Although the peer‐review system and the requirement to report conflicts of interest in published articles address some of these issues, these mechanisms are not entirely efficient.

Understanding of these two issues may elicit a more critical attitude to scientific news, prompt more consideration of potential benefits and harms of sharing a given content—as well as the use of social media in general—so that it is not based on compulsive reactions but is thoughtful and aligned with one's goals and values. Prudence in using the Internet, including critical attitude towards information, should be inculcated as early as possible by parents and teachers, since the young may be more prone to fall prey to the strategies employed by the online communication platforms. What is related to this, efforts should be taken to introduce classes tackling these topics in school and university curricula as well as to develop research to increase understanding of the problems related to the use of social media, including the issue of misinformation, and its impact on society.

Prudence in using the Internet, including critical attitude towards information, should be inculcated as early as possible …

Although the censorship on social media may seem an efficient and immediate solution to the problem of medical and scientific misinformation, it paradoxically introduces a risk of propagation of errors and manipulation. This is related to the fact that the exclusive authority to define what is “scientifically proven” or “medically substantiated” is attributed to either the social media providers or certain institutions, despite the possibility of mistakes on their side or potential abuse of their position to foster political, commercial or other interests. Focusing on understanding and studying the problem of misinformation, education and promotion of a virtuous use of social media and information seem more laborious and may not bring immediate results, but, in the long run, may contribute to a society that is more immune to infodemics.

## Conflict of interest

The author declares no conflict of interest.
